# A year in pharmacology: new drugs approved by the US food and drug administration in 2025

**DOI:** 10.1007/s00210-026-05193-0

**Published:** 2026-03-11

**Authors:** Gizem Kayki-Mutlu, Zinnet Sevval Aksoyalp, Leszek Wojnowski, Martin C. Michel

**Affiliations:** 1 Department of Pharmacology, Faculty of Pharmacy, Ankara University, Ankara, Türkiye; 2 Department of Pharmacology, Faculty of Pharmacy, Izmir Katip Celebi University, Izmir, Türkiye; 3 Department of Pharmacology, University Medical Center, Johannes Gutenberg University, Mainz, Germany; 4 Pharmaceutical Medicine, Charlotte Fresenius University, Wiesbaden, Germany

**Keywords:** Cell-based therapy, First-in-class, Gene therapy, Monoclonal antibody, New drugs, Next-in-class, Orphan drugs, Rare diseases

## Abstract

The US Food and Drug Administration approved 45 new drugs and 4 new cellular and gene therapy products in 2025, that is, a total of 49 new medical therapies. Oncology, hematology/immunotherapy, and cardiovascular/respiratory drugs remain most frequent. Among oncology drugs, there is a trend for targeting ever smaller subtypes of malignancies and/or rarer malignancies. Trends over the past 6 years include steady levels of first-in-indication (about 10%), first-in-class (about 38%), and next-in-class (about 51%) approvals. Along with a large share of approvals in orphan indications (> 50%), fast track approval pathways are now regularly representing more than half of all new approvals. While small molecules remain the bedrock of novel medical treatments (about 60%), there is a growing segment of mRNA, gene, and cell-based pharmacological approaches. These data testify to the innovative strength of academia, biotechnology, and established pharmaceutical companies in providing innovative treatments, often related to previously unmet medical needs.

## Introduction

We have previously reviewed and analyzed new drug approvals by the US Food and Drug Administration (FDA) for 2020–2024 (Kayki-Mutlu and Michel 2021; Kayki-Mutlu et al. 2022, 2023, 2024; Aksoyalp et al. 2025). In this sixth part of our annual series, we analyze such approvals in 2025. Our data confirm a steady level of approximately 50 new approvals per year.

**Fig. 1:**
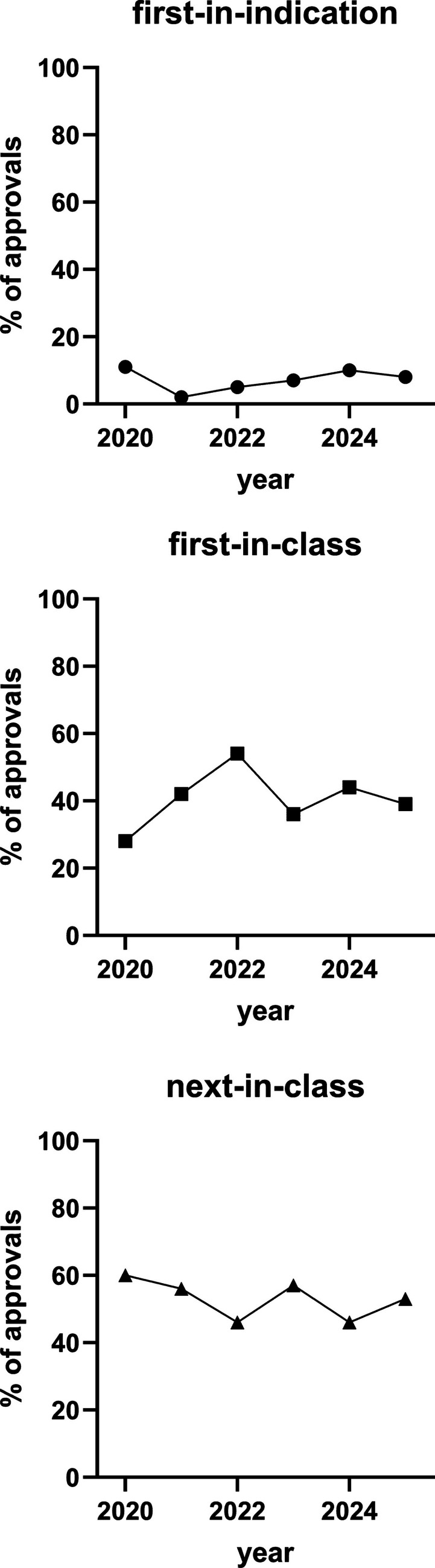
Degree of innovation in drugs approved in 2020–2025

**Fig. 2:**
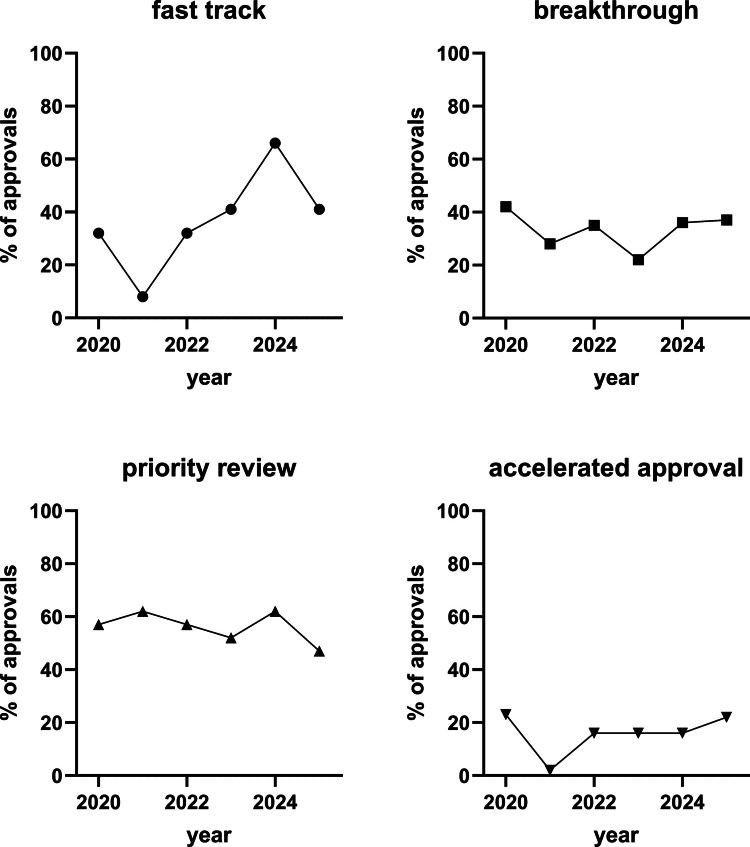
Approval paths for newly approved drugs in 2020–2025

**Fig. 3:**
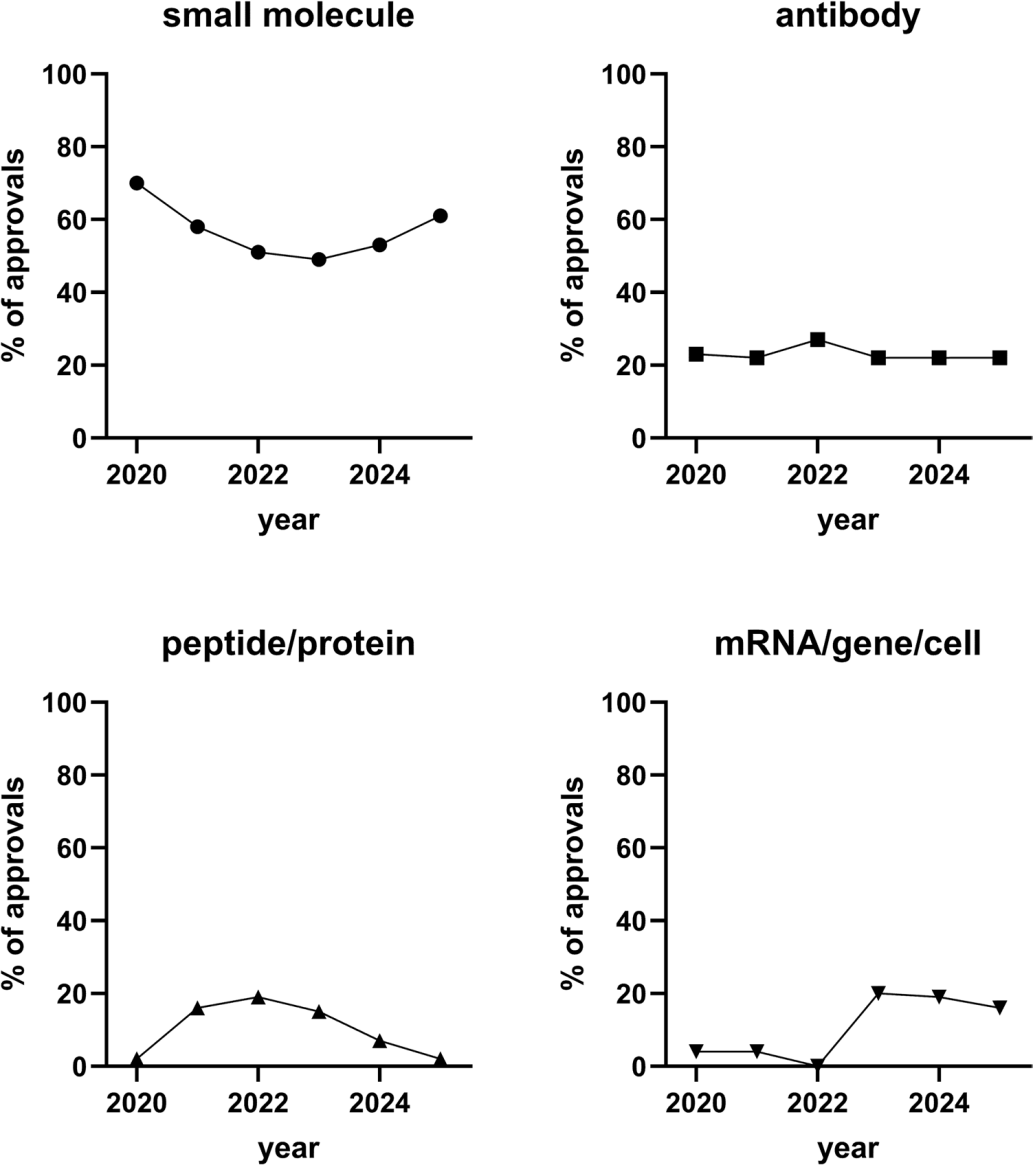
Drug types of newly approved drugs in 2020–2025

In line with earlier parts of the series, we briefly summarize efficacy and tolerability data for each new drug. The degree of innovation is classified as first-in-indication, that is, drugs for the treatment of a condition for which no approved medical treatments existed; first-in-class, that is, drugs with a molecular mechanism of action that had not been used by previously approved medical treatments; and next-in-class, that is, novel chemical or biological entities that exploit a molecular mechanism already available for the treatment of the same condition (Table 1 and Fig. 1). We also analyze new approvals by regulatory pathway (Fig. 2) and by molecular structure (small molecules, peptides and proteins, antibodies, and cellular and gene therapy; Table 2 and Fig. 3). Special attention is given to drugs assigned orphan status (Table 3). Finally, trends over the last 5 years are analyzed.

**Table 1:** Newly approved drugs grouped by novelty. For definitions, see “Introduction.” Percentages are those of first-in-indication, first-in-class, and next-in-class drugs with all drugs (including cellular and gene therapies) approved in 2025 taken as 100%. Cellular and gene products are depicted by italics. Where available, the International Nonproprietary Name stems in drug names have been highlighted by underlining based on the WHO Stem Book (https://cdn.who.int/media/docs/default-source/international-nonproprietary-names-(inn)/addendum-stembook2018-202402.pdf?sfvrsn=39190640_3&download=true; https://cdn.who.int/media/docs/default-source/international-nonproprietary-names-(inn)/inn-bio-review-2022.pdf?sfvrsn=f8db166f_3&download=true; https://cdn.who.int/media/docs/default-source/international-nonproprietary-names-(inn)/stembook-2018.pdf)

First-in-indication (*n* = 5, 10.2%)	Approved for	First-in-class (*n* = 19, 38.8%)	Approved for	Next-in-class (*n* = 25, 51%)	Approved for
Brensocatib	Noncystic fibrosis diseases	Acoltremon	Dry eye disease	Aficamten	Obstructive hypertrophic cardiomyopathy
Doxecitine and doxribtimine	Thymidine kinase 2 deficiency	Avutometinib and defactinib	KRAS-mutated recurrent low-grade serous ovarian cancer	Atrasentan	Primary IgA nephropathy
Elamipretide	Barth syndrome	Donidalorsen	Prophylaxis to prevent attacks of hereditary angioedema	Clesrovimab-cfor	Respiratory syncytial virus (RSV)
Narsoplimab-wuug	Hematopoietic stem cell transplant–associated thrombotic microangiopathy	Dordaviprone	Diffuse midline glioma harboring an H3 K27M mutation	Datopotamab deruxtecan-dlnk	Unresectable or metastatic, HR-positive, HER2-negative breast cancer
*Revakinagene-taroretcel*	Macular telangiectasia type 2	Elinzanetant	Vasomotor symptoms due to menopause	Delgocitinib	Chronic hand eczema
		*Etuvetidigene autotemcel*	Wiskott–Aldrich syndrome	Depemokimab-ulaa	Severe asthma
		Fitusiran	Hemophilia A or B	Etripamil	Paroxysmal supraventricular tachycardia
		Garadacimab-gxii	Prophylaxis to prevent attacks of hereditary angioedema	Imlunestrant	Estrogen receptor-positive, human epidermal growth factor receptor 2-negative, estrogen receptor-1-mutated advanced or metastatic breast cancer
		Gepotidacin	Uncomplicated urinary tract infections	Lerodalcibep-liga	Heterozygous familial hypercholesterolemia
		Nerandomilast	Pulmonary fibrosis	Linvoseltamab-gcpt	Relapsed or refractory multiple myeloma
		Plozasiran	Familial chylomicronemia syndrome	Mirdametinib	Neurofibromatosis
		*Prademagene-zamikeracel*	Recessive dystrophic epidermolysis bullosa	Nipocalimab-aahu	Myasthenia gravis
		Remibrutinib	Chronic spontaneous urticaria	Paltusotine	Acromegaly
		Rilzabrutinib	Persistent or chronic immune thrombocytopenia	Pembrolizumab and berahyaluronidase alpha-pmph	Multiple solid tumors
		Sibeprenlimab-szsi	Primary immunoglobulin A nephropathy	Penpulimab	Recurrent or metastatic nonkeratinizing nasopharyngeal carcinoma
		Suzetrigine	Moderate to severe acute pain	Sebetralstat	Acute attacks of hereditary angioedema
		Telisotuzumab vedotin-tllv	Locally advanced or metastatic, nonsquamous non–small cell lung cancer (NSCLC) with high c-Met protein overexpression	Sepiapterin	Hyperphenylalaninemia
		*Zopapogene imadenovec-drba*	Recurrent respiratory papillomatosis	Sevabertinib	Locally advanced or metastatic nonsquamous NSCLC that has HER2 (ERBB2) tyrosine kinase domain activating mutations
		Zoliflodacin	Uncomplicated urogenital gonorrhea	Sunvozertinib	Locally advanced or metastatic NSCLC with EGFR exon 20 insertion mutations
				Taletrectinib	Locally advanced or metastatic *ROS1*-positive NSCLC
				Tradipitant	Vomiting induced by motion
				Treosulfan	Preparative regimen for allogeneic hematopoietic stem cell transplantation for acute myeloid leukemia and myelodysplastic syndrome
				Vimseltinib	Symptomatic tenosynovial giant cell tumor
				Ziftomenib	Relapsed or refractory acute myeloid leukemia in adults carrying a susceptible *NPM1* mutation
				Zongertinib	Unresectable or metastatic nonsquamous NSCLC whose tumors have HER2 tyrosine kinase domain activating mutations

**Table 2:** Newly approved drugs grouped by drug type. Percentages are those of small molecules, antibody, peptide-protein drugs, cellular and gene therapies, with all drugs approved by the FDA in 2025 taken as 100%. Cellular and gene products are depicted by italics

Small molecules (*n* = 30, 61.2%)	Antibody (*n* = 11, 22.4%)	Peptide and protein (*n* = 1, 2.1%)	Nucleic acid-based (*n* = 1, 2.1%)	Cellular and gene products (*n* = 6, 12.2%)
Acoltremon	Clesrovimab-cfor	Lerodalcibep	Donidalorsen	*Etuvetidigene autotemcel*
Aficamten	Datopotamab deruxtecan-dlnk			Fitusiran
Atrasentan	Depemokimab-ulaa			Plozasiran
Avutometinib and defactinib	Garadacimab-gxii			*Prademagene zamikeracel*
Brensocatib	Linvoseltamab-gcpt			*Revakinagene taroretcel-lwey*
Delgocitinib	Narsoplimab-wuug			*Zopapogene imadenovec-drba*
Dordaviprone	Nipocalimab-aahu			
Doxecitine and doxribtimine	Pembrolizumab and berahyaluronidase alpha-pmph			
Elamipretide	Penpulimab-kcqx			
Elinzanetant	Sibeprenlimab-szsi			
Etripamil	Telisotuzumab vedotin-tllv			
Gepotidacin				
Imlunestrant				
Mirdametinib				
Nerandomilast				
Paltusotine				
Remibrutinib				
Rilzabrutinib				
Sebetralstat				
Sepiapterin				
Sevabertinib				
Sunvozertinib				
Suzetrigine				
Taletrectinib				
Tradipitant				
Treosulfan				
Vimseltinib				
Ziftomenib				
Zoliflodacin				
Zongertinib

**Table 3:** Orphan drugs approved by the FDA in 2025. Percentage is that of orphan drugs within all drugs approved by the FDA in 2025 taken as 100%. Cellular and gene products are depicted in italics. https://www.accessdata.fda.gov/scripts/opdlisting/oopd/listResult.cfm

Drug (*n* = 28, 55%)	Major indications
Aficamten	Obstructive hypertrophic cardiomyopathy
Avutometinib and defactinib	KRAS-mutated recurrent low-grade serous ovarian cancer
Donidalorsen	Hereditary angioedema attacks
Dordaviprone	Diffuse midline glioma harboring an H3 K27M mutation
Doxecitine and doxribtimine	Thymidine kinase 2 deficiency
Elamipretide	Barth syndrome
*Etuvetidigene autotemcel*	Wiskott–Aldrich syndrome (WAS) who have a mutation in the WAS gene
Fitusiran	Hemophilia A or B
Garadacimab-gxii	Hereditary angioedema attacks
Linvoseltamab-gcpt	Relapsed or refractory multiple myeloma
Mirdametinib	Neurofibromatosis type 1
Narsoplimab-wuug	Hematopoietic stem cell transplant–associated thrombotic microangiopathy
Nerandomilast	Idiopathic pulmonary fibrosis
Nipocalimab-aahu	Generalized myasthenia gravis
Paltusotine	Acromegaly
Penpulimab-kcqx	Recurrent or metastatic nonkeratinizing nasopharyngeal carcinoma
Plozasiran	Familial chylomicronemia syndrome
*Prademagene zamikeracel*	Recessive dystrophic epidermolysis bullosa
*Revakinagene taroretcel-lwey*	Idiopathic macular telangiectasia type 2
Rilzabrutinib	Persistent or chronic immune thrombocytopenia
Sebetralstat	Hereditary angioedema attacks
Sepiapterin	Hyperphenylalaninemia
Sevabertinib	Locally advanced or metastatic nonsquamous non–small cell lung cancer with tumors that have activating HER2 tyrosine kinase domain activating mutations
Taletrectinib	Locally advanced or metastatic ROS1-positive non–small cell lung cancer
Treosulfan	Preparative regimen for allogeneic hematopoietic stem cell transplantation for acute myeloid leukemia and myelodysplastic syndrome
*Zopapogene imadenovec-drba*	Respiratory papillomatosis
Ziftomenib	Relapsed or refractory acute myeloid leukemia with a susceptible nucleophosmin 1 mutation

## Methods

Our analyses follow the same protocol as those for newly approved drugs in 2020–2024 (Kayki-Mutlu and Michel 2021; Kayki-Mutlu et al. 2022, 2023, 2024; Aksoyalp et al. 2025). Thus, we do not consider generics or generic versions of biopharmaceuticals (“biosimilars”) and already approved drugs that received marketing authorizations for one or more additional indications and/or in a novel formulation. Newly approved drug combinations were only considered if at least one of the combination partners (mostly therapeutic antibodies) is a novel chemical or biopharmaceutical entity. We would like to emphasize that other regulatory agencies may have approved the same compounds earlier than the FDA (among this year’s approvals, e.g., treosulfan in the European Union, datopotamab in the European Union and Japan, penpulimab and sunvozertinib in China), may do so at later points in time, may choose not to approve some of these compounds, or may choose to approve compounds not approved by the FDA. Our focus on drug approvals by the FDA does not imply any opinion on the scientific quality of approvals by the FDA as compared to the regulatory authorities in other jurisdictions but rather uses the FDA as a point of reference due to its status as one of the most influential drug regulatory authorities. All indications refer to adults unless stated otherwise.

We emphasize that we do not compare the specific advantages and disadvantages of novel treatments with available treatments, because this is best done by experts in a specific therapeutic area. Similarly, we do not discuss drug pricing for novel treatments: Such discussion can only be meaningful based on input from experts within a specific therapeutic area who can judge on the added clinical value of a treatment. They fall into the responsibility of health technology assessment bodies such as the National Institute for Health and Care Excellence in the United Kingdom.

## Oncology

In 2025, the FDA approved two drugs for the treatment of breast cancer: *datopotamab deruxtecan-dlnk* and *imlunestrant*. *Datopotamab deruxtecan-dlnk* was approved for the treatment of unresectable or metastatic, hormone receptor (HR)-positive, human epidermal growth factor receptor 2 (HER2)–negative breast cancer in women who have received prior endocrine-based therapy and chemotherapy for unresectable or metastatic disease (Royce et al. 2025). This has been granted accelerated approval. Datopotamab deruxtecan had previously been approved in the European Union and Japan (Blair 2025b). It is an antibody–drug conjugate (ADC) and consists of a trophoblast cell surface antigen 2 (TROP2)–directed humanized monoclonal antibody, a topoisomerase I inhibitor, and a tetrapeptide-based cleavable linker (Blair 2025b). Datopotamab deruxtecan binds to TROP2, which is located on the surface of tumor cells. Following internalization, the binding site is then subject to degradation by lysosomal enzymes (Okajima et al. 2021). The released topoisomerase inhibitor leads to the DNA damage and apoptosis of tumor cells (Okajima et al. 2021). Warnings and precautions include the potential for pulmonary and ocular problems, pneumonitis, ocular adverse events (AEs), oral sores, and embryo-fetal toxicity (Blair 2025b). The most frequent AEs were nausea, stomatitis, hair loss, tiredness, dry eye, and vomiting (Blair 2025b). Another medication that received approval for the treatment of breast cancer in 2025 is *imlunestrant,* a next-generation, oral, selective estrogen receptor (ER) degrader acting as a pure ER antagonist (Keam 2025b). Imlunestrant was approved for the treatment of ER-positive, HER2-negative, *ESR1*-mutated advanced or metastatic breast cancer in adults with disease progression following at least one line of endocrine therapy (Keam 2025b). This has been granted fast track designation. It binds specifically to the ER, leading to its degradation and, consequently, the downregulation of ER-dependent genes and suppression of cell growth (Sherman et al. 2025). Warnings and precautions include embryo-fetal toxicity; the most frequent AEs were decreased hemoglobin, calcium, neutrophils, and platelet levels; increased liver enzymes, cholesterol, and triglycerides; pain; tiredness; and gastrointestinal problems (Keam 2025b).

Neurofibromatosis type 1 or von Recklinghausen syndrome is a rare and progressive autosomal dominant tumor predisposition syndrome that causes the formation of plexiform neurofibromas, with the potential to result in significant morbidity (Oztosun et al. 2025, Saara and Rani 2025). *Mirdametinib* has been indicated as next-in-class agent in adult and pediatric patients with neurofibromatosis type 1 who have symptomatic plexiform neurofibromas and inoperable tumors (Hoy 2025a). This has been granted priority review and fast track designation. It is a selective, potent mitogen-activated protein kinase kinase (MEK) 1 and 2 inhibitor (Hoy 2025a; Oztosun et al. 2025) and regulates the Ras-mitogen–activated protein kinase (MAPK) signaling pathway, which is overactive in neurofibromatosis type 1-associated tumors (Saara and Rani 2025). Selumetinib, the first MEK inhibitor approved by the FDA in 2020, is indicated for the diagnosis of inoperable plexiform neurofibromas in children with neurofibromatosis type 1 (Saara and Rani 2025). Mirdametinib has warnings and precautions regarding cardiovascular, dermatologic, and ocular adverse reactions and embryo-fetal toxicity. The most frequent AEs were tiredness and dermatologic, cardiovascular, and gastrointestinal problems (Hoy 2025a).

*Penpulimab-kcqx* is a humanized immunoglobulin G1 monoclonal antibody that blocks programmed death receptor-1 (PD-1) and acts as an immune checkpoint inhibitor (Dhillon 2021). It is an immunotherapeutic agent that binds to the PD-1 receptor, thereby preventing interaction between tumor and T cells (Hockett et al. 2025). Consequently, this results in a reduction in tumor growth by reversing the inhibition of the PD-1-mediated immune response. In 2021, penpulimab was first approved in China for the treatment of relapsed or refractory classic Hodgkin’s lymphoma in adults (Dhillon 2021). In 2025, penpulimab was approved by the FDA for recurrent or metastatic nonkeratinizing nasopharyngeal carcinoma both as first-line therapy in combination with cisplatin or carboplatin and gemcitabine and as monotherapy (Umrani 2025). Penpulimab has been granted fast track designation, breakthrough designation, and orphan drug designation. Warnings and precautions comprise immune-mediated and infusion-related adverse reactions, transplant-related complications, and embryo-fetal toxicity. The most common AEs were gastrointestinal, pulmonary, and dermatologic problems; hypothyroidism; anemia; tiredness; fever; reduced appetite; and weight.

The combination of the PD-1 inhibitor *pembrolizumab* with *berahyaluronidase alpha-pmph*, an endoglycosidase, has also received FDA approval for administration via subcutaneous injection. As subcutaneous administration is a less complex process than intravenous administration, the subcutaneous administration has the advantage of enhancing patient satisfaction and thereby improving clinical efficacy (Cohen et al. 2025; Felip et al. 2025; Song et al. 2025). This combination has been approved for use in adult and pediatric patients (aged ≥ 12 years) for the treatment of solid tumors, for which the intravenous formulation of pembrolizumab is indicated. This has been granted accelerated approval. Pembrolizumab, a monoclonal antibody for the PD-1 receptor, was granted its initial approval by the FDA in 2014 for the treatment of patients with advanced or inoperable melanoma who failed to respond to other medications (Poole 2014) and received multiple additional oncological indications thereafter. *Berahyaluronidase alpha-pmph*, through the reversible and local degradation of hyaluronan in the extracellular matrix of the subcutaneous tissue, is combined with pembrolizumab to increase its permeability and distribution (Felip et al. 2025). The prescribing information includes warnings and precautions for immune-mediated AEs, hypersensitivity and administration-related reactions, complications of allogeneic hematopoietic stem cell transplantation, and embryo-fetal toxicity. The most common AEs were pain, nausea, and tiredness.

Low-grade serous ovarian cancer is a rare malignancy, most commonly observed in younger women, and Kirsten rat sarcoma virus homolog (*KRAS*) mutation is prevalent among these patients (Banerjee et al. 2025; Stover et al. 2025). A combination of *avutometinib* and *defactinib* was approved for the treatment of *KRAS*-mutated recurrent low-grade serous ovarian cancer after receiving prior systemic therapy (Blair 2025a). This has been granted accelerated approval, priority review, breakthrough therapy, and orphan drug designation. Avutometinib is a first-in-class, oral RAF/MEK inhibitor, and defactinib is a focal adhesion kinase (FAK)/proline-rich tyrosine kinase-2 (Pyk2) inhibitor (Banerjee et al. 2025; Blair 2025a). RAF and MEK proteins are involved in a signaling pathway that regulates processes such as cell proliferation, differentiation, survival, and migration (McCubrey et al. 2007). Avutometinib prevents the phosphorylation of MEK1/2 by RAF; however, this results in compensatory increased FAK activation, leading to anticancer drug resistance (Banerjee et al. 2025). Therefore, a co-packaging of avutometinib with defactinib, a FAK inhibitor, has been approved to prevent the resistance development. This combination has warnings and precautions regarding rhabdomyolysis, ocular, dermatologic, liver, and embryo-fetal toxicity. The most common treatment-related AEs were gastrointestinal disorders, anemia, abnormal liver enzymes, dermatologic problems, tiredness, swelling, and visual impairments (Blair 2025a).

The FDA approved five drugs for the treatment of non–small cell lung cancer (NSCLC) in 2025. *Telisotuzumab vedotin-tllv* is a first-in-class ADC that consists of a c-mesenchymal-epithelial transition factor (c-MET)–directed humanized antibody and microtubule inhibitor (Blair 2025e; Zhao et al. 2025). The monoclonal antibody is bound to monomethylauristatin E, a microtubule disruptor, via a cleavable linker (Wang et al. 2017). The ADC binds to c-Met receptors present on the surface of tumor cells and is subsequently internalized. The released monomethylauristatin E inhibits microtubule polymerization, induces cell cycle arrest, and promotes apoptosis (Wang et al. 2017). It has been approved for the treatment of locally advanced or metastatic, nonsquamous NSCLC with high c-Met protein overexpression after prior systemic therapy (Blair 2025e). This was granted accelerated approval, priority review, and breakthrough therapy designation. Telisotuzumab vedotin includes warnings and precautions about pulmonary, ocular, and neurologic problems; infusion-related reactions; and embryo-fetal toxicity (Blair 2025e). Most frequent treatment-related AEs were tiredness, swelling, reduced appetite, peripheral neuropathy, and laboratory abnormalities (Blair 2025e). *Taletrectinib* was also approved for the treatment of NSCLC, specifically to treat locally advanced or metastatic *ROS1*-positive NSCLC in adults (Hoy 2025c). It is a *proto-oncogene tyrosine-protein kinase-1* (*c-Ros oncogene-1; ROS1*) inhibitor, also found to inhibit tropomyosin receptor kinases (TRK)A, TRKB, and TRKC (Hoy 2025c). Taletrectinib acts by binding to ROS1 kinase, thereby inhibiting its signaling pathways and thus suppressing the tumor growth (Zahra et al. 2025). Taletrectinib is superior to older generation tyrosine kinase inhibitors in its capacity to overcome resistance mutations and control brain metastasis (Khan et al. 2025; Zahra et al. 2025). This was granted priority review, breakthrough designation, and orphan drug designation. Its warnings and precautions include pulmonary, cardiovascular, liver, and musculoskeletal problems and embryo-fetal toxicity. Taletrectinib has been reported to have a favorable tolerability and safety profile (Perol et al. 2025). The most frequent treatment-related AEs were gastrointestinal problems and laboratory abnormalities (Hoy 2025c). *Sunvozertinib* is an epidermal growth factor receptor (EGFR) tyrosine kinase inhibitor that binds to and irreversibly inhibits EGFR exon 20 insertion mutations (Dhillon 2023). EGFR is involved in the activation of signaling pathways that control cell proliferation, survival mechanisms, and differentiation, and its mutations can lead to dysregulated cell proliferation and tumor progression (Sangwan and Agarwal 2025). Sunvozertinib selectively binds to the mutant EGFR form, thereby inhibiting its kinase activity and preventing the activation of signaling pathways that promote tumor growth and progression (Wang et al. 2025b). In 2023, this drug received the first approval for the treatment of NSCLC in China (Dhillon 2023). In 2025, the FDA approved sunvozertinib for the treatment of locally advanced or metastatic NSCLC with EGFR exon 20 insertion mutations in adults with disease that has progressed on or following platinum-based chemotherapy (Sangwan and Agarwal 2025). This was granted priority review, accelerated approval, and breakthrough therapy designation. Warnings and precautions include pulmonary disorders, gastrointestinal and dermatologic adverse reactions, and ocular and embryo-fetal toxicity. The most frequent AEs were laboratory abnormalities, decline in appetite, and gastrointestinal and dermatologic problems (Dhillon 2023). Lastly, *zongertinib* and *sevabertinib* have been approved for the treatment of nonsquamous NSCLC harboring HER2 tyrosine kinase domain activating mutations in adults following treatment with systemic therapy. For cases of unresectable or metastatic nonsquamous NSCLC, zongertinib is indicated, while sevabertinib is reserved for locally advanced or metastatic nonsquamous NSCLC. Zongertinib and sevabertinib were granted priority review, accelerated approval, breakthrough therapy designation, and orphan drug designation. Zongertinib has also been granted fast track designation. *Zongertinib* is an oral, selective, and irreversible tyrosine kinase inhibitor for tumors with HER2 mutations, while sparing wild-type EGFR (Brazel et al. 2025; Trillo Aliaga et al. 2025). It exerts its anti-tumor efficacy by inhibiting HER2 phosphorylation and the proliferation of lung cancer cells. This drug has warnings and precautions regarding cardiovascular, hepatic, and pulmonary disorders and embryo-fetal toxicity. The most common treatment-related AEs were laboratory abnormalities and gastrointestinal and dermatologic problems (Brazel et al. 2025). *Sevabertinib* is an oral, potent, and reversible dual tyrosine kinase inhibitor for EGFR and HER2 mutations, including exon 20 insertion (Le et al. 2025; Siegel et al. 2025). It is also selective with respect to wild-type EGFR (Siegel et al. 2025). Sevabertinib includes warnings and precautions about diarrhea, pulmonary disease, increased pancreatic enzymes, liver, ocular, and embryo-fetal toxicity. The most common AEs were diarrhea, rash, paronychia, and nausea (Le et al. 2025).

Multiple myeloma is a hematological malignancy of mature B cells incurable by current treatments (Bumma et al. 2024; Lee 2025b). *Linvoseltamab-gcpt* is a human B cell maturation antigen (BCMA)–directed cluster of differentiation (CD)3 bispecific antibody (Ur Rehman 2025). It binds to the CD3 receptor on T cells and BCMA on multiple myeloma cells, and this engagement leads to the activation of T cells and the subsequent targeted destruction of malignant plasma cells (Ur Rehman 2025). Linvoseltamab has been approved to treat relapsed or refractory multiple myeloma in adults following the administration of at least four prior lines of therapy (Ur Rehman 2025). It was granted accelerated approval, priority review, orphan drug designation, and fast track designation. This drug has warnings and precautions regarding infections, neutropenia, liver, and embryo-fetal toxicity. Linvoseltamab also has a black box warning about cytokine release syndrome and neurotoxicity. The most frequent AEs were cytokine release syndrome, pain, tiredness, anemia, neutropenia, pulmonary, and gastrointestinal problems (Lee 2025b).

Acute leukemia is a heterogeneous hematological malignancy and is most commonly found in the form of acute myeloid leukemia, with a mutation in the nucleophosmin 1 (*NPM1*) gene in approximately one-third of cases (Falini et al. 2005). *Ziftomenib* has been approved for the treatment of relapsed or refractory acute myeloid leukemia in adults carrying a susceptible *NPM1* mutation and with a lack of satisfactory alternative treatment options. This application received priority review, breakthrough, fast track, and orphan drug designations. Ziftomenib is an oral, highly selective, and potent menin (MEN1) inhibitor (Wang et al. 2025a). It inhibits the oncogenic activity of mutant *NPM1* and promotes the differentiation of leukemic cells by preventing the interaction between menin and lysine-specific methyltransferase 2 A (KMT2A) (Rausch et al. 2023). This drug has warnings and precautions regarding QT_c_ prolongation and embryo-fetal toxicity. Ziftomenib also has a black box warning about differentiation syndrome. The most common AEs were gastrointestinal problems, infection, edema, hypokalemia, pruritus, and hematologic AE (Wang et al. 2025a).

Diffuse midline glioma is a rare and highly aggressive form of glioma, predominantly impacting children and young adults and carrying a distinctive histone H3 mutation (H3 K27M) (Bibi et al. 2025a). In the case of H3 K27M mutant gliomas, surgical resection is generally unsuitable, with the standard treatment method based on radiotherapy (Arrillaga-Romany et al. 2024). *Dordaviprone* is the first-in-class drug to treat diffuse midline glioma in adult and pediatric patients with H3 K27M mutation and progressive disease after prior treatment (Blair 2025c). Dordaviprone is a mitochondrial caseinolytic protease P activator and a bitopic antagonist of dopamine receptor 2/3; thus, it induces apoptosis through activating the tumor necrosis factor–related apoptosis-inducing ligand pathway (Bibi et al. 2025a; Blair 2025c). It was granted accelerated approval, priority review, orphan drug designation, and rare pediatric disease designation (Blair 2025c). Dordaviprone includes warnings and precautions about hypersensitivity, QT_c_ prolongation, and embryo-fetal toxicity. The most frequent treatment-related AEs were laboratory abnormalities, gastrointestinal problems, tiredness, pain, reduced appetite, anemia, loss of balance, rash, and muscle weakness (Blair 2025c).

Tenosynovial giant cell tumor is a rare mesenchymal neoplasm that affects the synovium, bursae, and tendons of joints and is typified by genomic abnormalities, particularly in the *colony-stimulating factor 1* (*CSF1*) gene (Stacchiotti et al. 2023). The CSF1 receptor kinase inhibitor* vimseltinib* has been approved for the treatment of symptomatic tenosynovial giant cell tumor in adults for which surgical resection would potentially lead to a deterioration in functional ability or severe morbidity (Lee 2025d). It was granted priority review and fast track designation. Vimseltinib demonstrates a high binding affinity and selectivity for CSF1 receptors; this results in the stabilization of the inactive state of the receptors and the inhibition of cell growth (Caldwell et al. 2022; Lee 2025d). Vimseltinib includes warnings and precautions about allergic reactions, enhanced serum creatinine levels, and liver and embryo-fetal toxicity. Most common AEs were edema, tiredness, rash, and pruritus (Lee 2025d).

## Hematology/immunology

In hematopoietic stem cell transplantation for hematological malignancies, preparative or conditioning regimens are utilized to prevent graft rejection and reduce tumor burden (Gyurkocza and Sandmaier 2014). The standard conditioning regimen typically consists of high-dose systemic chemotherapy and/or whole-body irradiation; however, toxicities associated with this regimen impact treatment success (Nemecek et al. 2011). *Treosulfan*, a prodrug of an alkylating agent, has been approved for use in combination with fludarabine as a preparative regimen for allogeneic hematopoietic stem cell transplantation in patients (aged > 1 year) diagnosed with acute myeloid leukemia or myelodysplastic syndrome. Previously, this drug has been approved in Europe for the treatment of advanced ovarian cancer (Gyurkocza and Sandmaier 2014). It was granted orphan drug designation for ovarian cancer treatment and conditioning treatment prior to hematopoietic stem cell transplantation by the FDA. Treosulfan has a black box warning about myelosuppression. It includes warnings and precautions about seizures, injection-site reactions, secondary malignancies, and embryo-fetal toxicity. The most common AEs were gastrointestinal disorder, pain, fever, edema, and infection.

Hemophilia A and hemophilia B are genetic coagulation disorders characterized by impaired activity or absence of coagulation factor VIII or IX, respectively (Lu et al. 2025). The predominant therapeutic approach for hemophilia is replacement therapy with clotting factor concentrates; however, this treatment strategy has limitations (Marchesini et al. 2021). *Fitusiran* has been approved by the FDA for the routine prophylaxis of bleeding in adult and pediatric patients (aged ≥ 12 years) with hemophilia A or B (Lee 2025a). This application was granted orphan drug and fast track designations. A notable advantage of fitusiran is an extended dosing interval in comparison to replacement therapy (Lu et al. 2025). Fitusiran is an antithrombin-directed double-stranded small interfering RNA (siRNA) therapy that leads to the degradation of antithrombin mRNA and a consequent decrease in antithrombin and increase in thrombin levels. This drug has warnings and precautions regarding hepatotoxicity. Fitusiran also has a black box warning about thrombotic events and acute and recurrent gallbladder disease. Most frequent AEs were injection-site reactions; infections; pain; and respiratory, liver, and coagulation problems (Lee 2025a).

Hereditary angioedema is a rare genetic disorder characterized by dysfunction in the kallikrein-kinin cascade, leading to overproduction of bradykinin (Sinnathamby et al. 2023). In 2025, the FDA approved three drugs for the treatment of prophylaxis or acute attacks of hereditary angioedema: *garadacimab-gxii*, *sebetralstat*, and *donidalorsen.* Garadacimab-gxii and donidalorsen have been approved as first-in-class for the prophylaxis of hereditary angioedema, while sebetralstat has been approved for acute attacks in adults and pediatric patients (aged ≥ 12 years) (Blair 2025d; Fung 2025a; Syed 2025). Garadacimab-gxii, sebetralstat, and donidalorsen were granted orphan drug designation, and garadacimab-gxii and sebetralstat have also been granted fast track designation (Blair 2025d; Fung 2025a; Syed 2025). *Garadacimab-gxii* is a monoclonal antibody against activated factor XII (Fung 2025a). Factor XII activation leads to the generation of bradykinin, which is associated with inflammation and edema observed in hereditary angioedema attacks (Fung 2025a). Garadacimab-gxii binds to the catalytic domain of activated factor XII and thereby inhibits its catalytic activity (Fung 2025a). The inhibition of factor XIIa prevents hereditary angioedema attacks by reducing the activation of the prekallikrein-to-kallikrein cascade and bradykinin production (Beninger 2025; Fung 2025a). The prescription information does not include any warnings or precautions. The most common treatment-emergent AEs were upper respiratory tract infections, nasopharyngitis, injection-site reactions, and pain (Fung 2025a). *Donidalorsen* is an antisense oligonucleotide conjugated with triantennary N-acetylgalactosamine, which facilitates its transport to prekallikrein-producing hepatic cells (Syed 2025). It reduces the production of prekallikrein protein in the liver and decreases bradykinin production in patients with hereditary angioedema (Riedl et al. 2025). Donidalorsen has warnings and precautions about hypersensitivity reactions. The most frequent AEs were injection-site reactions, infections, and gastrointestinal distress (Syed 2025). Lastly, *sebetralstat* has been approved as a fast-acting, on-demand oral treatment option for hereditary angioedema attacks (Bibi et al. 2025b). It is a potent, competitive, and reversible orally self-administered plasma kallikrein inhibitor (Blair 2025d). Plasma kallikrein is involved in the degradation of high molecular weight kininogen, and sebetralstat, via its inhibitory effect on this process, results in a reduction of bradykinin production (Blair 2025d). In addition, sebetralstat inhibits the positive feedback mechanism of the kallikrein-kinin system, thereby decreasing factor XII activation and plasma kallikrein formation (Blair 2025d). The most frequent AEs were headache and flushing (Blair 2025d).

Immune thrombocytopenia is a rare, acquired autoimmune hematological disorder involving a series of immunological responses that result in decreased platelet levels and an augmented bleeding risk (Kuter and Ghanima 2025). *Rilzabrutinib* is a reversible, potent, highly specific Bruton’s tyrosine kinase (BTK) inhibitor and inhibits B cell proliferation, production of anti-platelet autoantibodies, and inflammatory pathways (Lee 2025c; Michel 2025). It is distinct from irreversible BTK inhibitors, with the potential to protect platelet function and reduce hemorrhagic risks (Labanca et al. 2025). Rilzabrutinib has been approved to treat persistent or chronic immune thrombocytopenia in adults in cases of inadequate response to prior treatment (Lee 2025c). This application received fast track designation. Warnings and precautions comprise infections and liver and embryo-fetal toxicity. Most frequent AEs were gastrointestinal problems, pain, and COVID-19 (Lee 2025c).

Transplantation-associated thrombotic microangiopathy is a prevalent complication of hematopoietic cell transplantation, marked by endothelial dysfunction, microthrombi formation, and dysregulation of the complement pathway (Chumnumsiriwath et al. 2025; Schoettler et al. 2025). *Narsoplimab-wuug* was approved for the treatment of hematopoietic stem cell transplant–associated thrombotic microangiopathy in adult and pediatric patients (aged ≥ 2 years). This application received priority review and breakthrough designation. Narsoplimab is a fully humanized monoclonal antibody that inhibits mannan-binding lectin-associated serine protease-2 (Chumnumsiriwath et al. 2025). The lectin pathway is effectively blocked with narsoplimab while the classical and alternative pathway of the complement system remains unaffected (Pandrowala et al. 2023). Therefore, lectin pathway–mediated endothelial damage is prevented by narsoplimab. Warnings and precautions include serious infections. The most common AEs were infections, gastrointestinal disorders, fever, tiredness, hemorrhage, and decreased potassium and neutrophil levels.

Wiskott–Aldrich syndrome is a rare, life-threatening, immunodeficiency disorder that is characterized by eczema, recurrent infections, and microthrombocytopenia in the early stages of life, due to loss-of-function mutations in the *WAS* gene (Rivers et al. 2019). The treatment of Wiskott–Aldrich syndrome involves hematopoietic stem cell transplantation or stem cell gene therapy (Rivers et al. 2019). *Etuvetidigene autotemcel*, an ex vivo gene therapy, has been approved for the treatment of Wiskott–Aldrich syndrome in pediatric patients (≥ 6 months) and in adults. The therapy consists of genetically modified autologous hematopoietic stem cells into which the *WAS*-corrected gene has been inserted, which enables the functional restoration of *WAS* protein expression (Sepodes et al. 2026). Patients eligible for this gene therapy include individuals with mutations in the *WAS* gene and suitable for hematopoietic stem cell transplantation but with no available compatible related stem cell donor with a matching human leukocyte antigen. This application has been granted orphan drug, advanced therapy, rare pediatric disease, and regenerative medicine designations. *Etuvetidigene autotemcel* has warnings and precautions regarding the potential for hypersensitivity and infusion-related reactions; engraftment failure; decreased blood cell counts; serious infections; infectious agent transmission; hepatic veno-occlusive disease; oncogenesis risk; interference with HIV testing; and restrictions on blood, organ, tissue, and cell donation in the prescription information. The most common AEs were infections; hepatic and head injury; anemia; febrile neutropenia; nosebleeds; fever; and gastrointestinal, dermatologic, and respiratory problems.

## Neurology

In contrast to previous years, in 2025, FDA approved only two new neurological drugs, suzetrigine and nipocalimab-aahu for the treatment of pain and myasthenia gravis, respectively. *Suzetrigine* was approved as a nonopioid treatment for moderate to severe acute pain in adults (Keam 2025c). It is a potent and selective inhibitor of voltage-gated sodium channel 1.8 (NaV1.8), which prevents the transmission of pain signals to the central nervous system (Osteen et al. 2025). Contrary to opioids, suzetrigine is considered nonaddictive, due to its inability to induce euphoria or excitement (Hu et al. 2025). This application received breakthrough therapy, fast track, and priority review designations. Suzetrigine has warnings and precautions about liver dysfunction. The most common AEs were itching, cramps, elevated creatine phosphokinase levels, and rash (Keam 2025c).

Myasthenia gravis is a chronic autoimmune disease triggered by the presence of pathogenic autoantibodies, leading to neuromuscular transmission disruption (Dresser et al. 2021). *Nipocalimab-aahu* was approved to treat generalized myasthenia gravis in adult and pediatric patients (aged ≥ 12 years) with positive anti-acetylcholine receptor or anti-muscle specific tyrosine kinase antibody tests (Fung 2025b). It is a human IgG1 monoclonal antibody that targets and blocks the neonatal fragment crystallizable (Fc) receptor (Antozzi and Fitzgibbon 2025). Consequently, this results in a decline in serum IgG, along with pathogenic acetylcholine receptor and muscle-specific kinase autoantibody levels (Antozzi and Fitzgibbon 2025; Seth et al. 2025). This application was granted orphan drug, fast track, and priority review designation (Fung 2025b). Nipocalimab has warnings and precautions about infusion-related reactions, hypersensitivity reactions, and infections. The most frequent AEs were respiratory tract infection, swelling, and cramps (Fung 2025b).

## Cardiovascular and respiratory disorders

*Elamipretide*, a mitochondrial cardiolipin-binding peptide, is approved for the treatment of Barth syndrome and represents the first therapy for this rare and life-threatening genetic disorder. Elamipretide targets cardiolipin, a phospholipid of the inner mitochondrial membrane, which improves mitochondrial morphology and function. Daily subcutaneous administration increases muscle strength. Clinical trials using descriptive and correlational analyses demonstrate improvements in symptom severity, fatigue, and myopathy scores (Gwaltney et al. 2025). Elamipretide has received priority review, orphan drug, fast track designations, and accelerated approval. Long-term treatment has been shown to be well tolerated, with sustained improvements in functional outcomes and cardiac function (Thompson et al. 2024). Injection-site reactions were reported.

*Etripamil* is approved for the treatment of acute symptomatic episodes of paroxysmal supraventricular (PSVT) tachycardia. It is the first self-administered intranasal therapy for this indication. Etripamil is a fast-acting, short-duration L-type calcium channel blocker. It effectively converted PSVT to sinus rhythm during both single and repeated episodes (Stambler et al. 2023; Ip et al. 2025). It is considered to be safe and well tolerated (Stambler et al. 2023). Administration is recommended in a seated position due to the risk of dizziness or syncope. The most common AEs include nasal discomfort, nasal congestion, rhinorrhea, throat irritation, and epistaxis. Etripamil is contraindicated in patients with heart failure and specific conduction disorders, including Wolff–Parkinson–White or Lown–Ganong–Levine syndrome.

The next-in-class cardiac myosin inhibitor *aficamten* was approved for the treatment of symptomatic obstructive hypertrophic cardiomyopathy in adults. It selectively targets β-cardiac myosin to reduce excessive actin–myosin cross-bridge formation and left ventricular contractility. Oral aficamten improves exercise capacity as demonstrated by increased peak oxygen uptake measured by cardiopulmonary exercise testing. Secondary endpoints including the New York Heart Association class, resting and Valsalva gradients, and NT-proBNP were also significantly improved (Maron et al. 2025). Treatment was associated with favorable cardiac remodeling, including reductions in left ventricular mass, wall thickness, and left atrial size, suggesting potential long-term cardiovascular benefit (Masri et al. 2024). Aficamten carries a boxed warning for heart failure due to reduced left ventricular ejection fraction, and hypertension is a commonly reported AE. The drug has received orphan drug and breakthrough therapy designations.

The FDA has approved *brensocatib* as the first and only treatment for noncystic fibrosis bronchiectasis, a chronic and progressive inflammatory lung disease characterized by permanent bronchi dilation. Brensocatib is an orally administered dipeptidyl peptidase-1 (DPP1) inhibitor that targets neutrophil serine proteases—key mediators of airway inflammation. Treatment has also been shown to reduce serine protease activity and improve clinical outcomes (Chalmers et al. 2020). Brensocatib also lowers the annualized rate of pulmonary exacerbations and improves forced expiratory volume in 1-s decline (Chalmers et al. 2025). Dermatological AEs include rash, dry skin, and hyperkeratosis, along with other common events such as respiratory tract infection, headache, and hypertension.

Another first-in-class drug, *nerandomilast*, is indicated for the treatment of idiopathic pulmonary fibrosis, a rare and progressive fibrotic lung disease. Nerandomilast is an oral phosphodiesterase-4 (PDE4) inhibitor with anti-fibrotic and immunomodulatory properties. Clinical trials demonstrated a reduced decline in forced vital capacity (FVC) over 52 weeks (Maher et al. 2025; Richeldi et al. 2025). Nerandomilast has received orphan drug, breakthrough therapy, and priority review designations. Common AEs include diarrhea, COVID-19, upper respiratory tract infection, depression, weight decrease, decreased appetite, nausea, fatigue, headache, vomiting, back pain, and dizziness.

The humanized IgG1 monoclonal antibody, *depemokimab-ulaa*, is approved as add-on maintenance therapy for severe asthma with an eosinophilic phenotype. It is a long-acting interleukin-5 antagonist that suppresses eosinophil production and activation (Nolasco and Crimi 2024). Subcutaneous administration every 6 months for 52 weeks reduced blood eosinophil counts in adult and pediatric patients (Jackson et al. 2024). Depemokimab was also well tolerated and reduced treatment burden in patients with chronic rhinosinusitis with nasal polyps (Gevaert et al. 2025). Upper respiratory tract infection, allergic rhinitis, influenza, arthralgia, and pharyngitis are among the most common AEs.

## Metabolic, endocrine, and hepatobiliary diseases

*Sepiapterin* is indicated for the treatment of hyperphenylalaninemia in adults and children aged ≥ 1 month with phenylketonuria in conjunction with a phenylalanine-restricted diet. It is a phenylalanine hydroxylase activator that reduces blood phenylalanine levels, is administered orally once daily, and received orphan drug designation. Sepiapterin produces a clinically meaningful reduction in blood phenylalanine levels and is generally well tolerated (Muntau et al. 2024). Therapy may increase the risk of bleeding and hypophenylalaninemia. Diarrhea, headache, abdominal pain, hypophenylalaninemia, feces discoloration, and oropharyngeal pain are among the common AEs.

A somatostatin receptor agonist, *paltusotine*, is approved to treat acromegaly in adults who have not achieved an adequate response to surgery or for whom surgery is not an option. It is the first once-daily oral therapy offering an alternative to monthly depot injections and received orphan drug designation. Transitioning patients with acromegaly from injectable somatostatin receptor ligands to once-daily oral paltusotine maintained biochemical control and was well tolerated. Hormone levels rise after paltusotine treatment withdrawal, supporting its effectiveness (Gadelha et al. 2023). Oral paltusotine demonstrates high bioavailability, efficient absorption, biliary excretion largely as unchanged drug, a half-life compatible with once-daily dosing, and good tolerability (Luo et al. 2025). Warnings and precautions include cholelithiasis, dysglycemia, cardiovascular effects, thyroid dysfunction, steatorrhea with fat malabsorption, and vitamin B12 deficiency. Frequently reported AEs include diarrhea, abdominal pain, nausea, decreased appetite, sinus bradycardia, hyperglycemia, palpitations, and gastroenteritis.

The nonhormonal, dual neurokinin 1 and 3 receptor antagonist *elinzanetant* was approved for the treatment of moderate to severe vasomotor symptoms due to menopause. Elinzanetant is a first-in-class, orally administered drug that modulates estrogen-sensitive hypothalamic neurons involved in thermoregulation. Elinzanetant reduces vasomotor symptoms and improves sleep and quality of life with good overall tolerability (Simon et al. 2023; Panay et al. 2025). In a phase 3 trial, elinzanetant decreased vasomotor symptoms in women receiving endocrine therapy for hormone receptor–positive breast cancer or for breast cancer prevention (Cardoso et al. 2025). Warnings and precautions include somnolence, elevations in hepatic transaminases, and risk of pregnancy loss. The most common AEs are headache, fatigue, dizziness, and somnolence.

Two drugs targeting dyslipidemia were approved. The apolipoprotein C-III (apoC-III)-directed siRNA *plozasiran* was approved for familial chylomicronemia syndrome to reduce triglyceride levels in adults with this condition. Silencing apoC-III relieves inhibition of lipoprotein lipase and enhances hepatic clearance of triglyceride-rich lipoproteins, resulting in sustained reductions in triglycerides, very-low-density lipoprotein, and chylomicrons. Plozasiran reduces triglyceride levels and the incidence of pancreatitis (Watts et al. 2025), with a favorable safety profile (Ballantyne et al. 2024; Gaudet et al. 2024). It is administered subcutaneously once every 3 months as an adjunct to diet. Common AEs include hyperglycemia, headache, nausea, and injection-site reactions. Plozasiran has received orphan drug, breakthrough therapy, and fast track designations.

The proprotein convertase subtilisin kexin type 9 (PCSK9) inhibitor *lerodalcibep-liga* was approved for the treatment of hypercholesterolemia in adults as an adjunct to diet and exercise. PCSK9 promotes degradation of hepatic low-density lipoprotein receptors, thereby regulating circulating LDL cholesterol levels (Corsini et al. 2025). Lerodalcibep binds PCSK9, preventing its interaction with LDL receptors and subsequent receptor degradation, thereby increasing hepatic LDL receptor availability and reducing circulating LDL cholesterol levels. Monthly subcutaneous lerodalcibep injections in patients with heterozygous familial hypercholesterolemia caused substantial mean reductions in LDL cholesterol (Raal et al. 2023, 2025). An LDL-C lowering was also observed in patients with cardiovascular disease or at high cardiovascular risk (Klug et al. 2024). Treatment was generally well tolerated, with AEs including injection-site reactions, nasopharyngitis, diarrhea, nausea, and peripheral edema.

## Renal disorders

Two drugs were approved in 2025 for the treatment of nephropathy, specifically targeting proteinuria in high-risk IgA nephropathy. The first is *atrasentan*, a selective endothelin type A receptor antagonist, indicated to reduce proteinuria in adults with primary IgA nephropathy who are at risk of rapid progression, defined by a urine protein-to-creatinine ratio ≥ 1.5 g/g. IgA nephropathy is driven by the formation of galactose-deficient, IgA-containing immune complexes, which trigger renal inflammation and fibrosis, as well as endothelin-1–mediated podocyte injury, ultimately leading to proteinuria and progressive loss of renal function (Keam 2025a). Oral atrasentan reduced urinary protein excretion and was generally well tolerated (Heerspink et al. 2025). It also reduced the risk of renal events in patients with diabetes and chronic kidney disease (Smeijer et al. 2025). The most commonly reported AEs were peripheral edema and anemia. Atrasentan carries a boxed warning for birth defects and is therefore contraindicated during pregnancy. The drug received accelerated approval, with the most frequently reported adverse effects being peripheral edema and anemia.

A second, first-in-class therapy, *sibeprenlimab-szsi*, was also approved for the treatment of proteinuria in adults with primary IgA nephropathy at risk for disease progression. Sibeprenlimab is the first monoclonal antibody targeting a proliferation-inducing ligand (APRIL), a member of the tumor necrosis factor superfamily that plays a central role in B cell activation and survival. Elevated APRIL levels have been observed in inflammatory conditions, including IgA nephropathy (Mathur et al. 2023). Early clinical studies demonstrated that sibeprenlimab produced dose-dependent and reversible suppression of APRIL and was well tolerated, with no serious AE reported (Mathur et al. 2022). Monthly intravenous sibeprenlimab reduced proteinuria in adults with high-risk IgA nephropathy (Mathur et al. 2024). Subcutaneous administration also reduced proteinuria in adults with biopsy-confirmed IgA nephropathy, which was associated with reductions in circulating IgA and IgM levels (Perkovic et al. 2026). Sibeprenlimab is additionally being evaluated for the treatment of Sjögren’s disease (Mullard 2026). The drug was generally well tolerated, with common AEs including upper respiratory tract infections and injection-site erythema. Sibeprenlimab received accelerated approval and was granted breakthrough therapy and priority review designations.

## Infectious diseases

Several first-in-class and targeted therapies for the treatment and prevention of infectious diseases were approved in 2025. *Gepotidacin* was approved for the treatment of uncomplicated urinary tract infections caused by *Escherichia coli*, *Klebsiella pneumoniae*, *Citrobacter freundii complex*, *Staphylococcus saprophyticus*, and *Enterococcus faecalis* in female patients aged 12 years and older and weighing at least 40 kg. In addition, gepotidacin is also indicated for the treatment of uncomplicated urogenital gonorrhea in patients with limited or no alternative treatment options. This first-in-class drug is a triazaacenaphthylene antibacterial agent that inhibits bacterial type II topoisomerases, specifically DNA gyrase and topoisomerase IV, thereby blocking bacterial DNA replication. Orally administered gepotidacin demonstrated efficacy comparable to or greater than nitrofurantoin, with generally mild to moderate AEs, most commonly diarrhea and nausea (Scangarella-Oman et al. 2025; Wagenlehner et al. 2025). In urogenital gonorrhea, gepotidacin showed noninferior efficacy compared with ceftriaxone plus azithromycin, while offering the practical advantage of an oral regimen and no new safety concerns (Ross et al. 2025). Importantly, gepotidacin is one of the few new therapeutic options against gonorrhea, including many rapidly expanding strains resistant to older drugs. Warnings and precautions of the therapy include QTc prolongation, acetylcholinesterase inhibition, hypersensitivity, and *Clostridioides difficile* infection. Gepotidacin received priority review designation.

Another advance urogenital gonorrhea therapy is *zoliflodacin*, a first-in-class spiropyrimidinetrione inhibitor of bacterial type II topoisomerase. Zoliflodacin has demonstrated potent in vitro activity against multidrug-resistant *N. gonorrhoeae*, including strains resistant to ceftriaxone and azithromycin, with no observed cross-resistance (Yao et al. 2026). Zoliflodacin showed noninferior efficacy compared with ceftriaxone plus azithromycin and a comparable safety profile, supporting its use as a single-dose oral treatment option (Luckey et al. 2026). Warnings and precautions include embryo-fetal toxicity, testicular toxicity and impairment of male fertility, hypersensitivity reactions, and *Clostridioides difficile* infection. Frequently reported AEs included neutropenia, leukopenia, headache, dizziness, nausea, and diarrhea. Zoliflodacin received fast track and priority review designations.

In contrast to small-molecule antibacterials, *clesrovimab-cfor* is a monoclonal antibody targeting respiratory syncytial virus (RSV) fusion protein. The drug was approved for the prevention of RSV lower respiratory tract disease in neonates and infants born during or entering their first RSV season. A single intramuscular dose of clesrovimab reduced RSV-associated lower respiratory infections and hospitalizations, with no meaningful safety differences versus placebo (Zar et al. 2025). Approval was further supported by a comparative trial demonstrating similar safety and efficacy to monthly intramuscular palivizumab in infants at increased risk for severe RSV disease (Sinha et al. 2025). Hypersensitivity reactions, including anaphylaxis, may occur, and the most common AEs were injection-site erythema, swelling, and rash.

Beyond acute infections, a novel gene therapy has been approved for a chronic virus–driven disease. *Zopapogene imadenovec-drba* was approved for the treatment of adult patients with recurrent respiratory papillomatosis. This is a rare disease caused by persistent human papillomavirus and characterized by recurrent benign tumors in the respiratory tract. *Zopapogene imadenovec* is the first nonreplicating adenoviral vector–based immunotherapy expressing an HPV-6/11 derived fusion antigen, designed to elicit a targeted immune response against HPV proteins (Lee 2026). It is currently the only FDA-approved therapy approved for the condition. The treatment is administered via subcutaneous injections four times over a 12-week period. Fifty-one percent of the treated patients achieved a complete response, and AEs were generally mild, most commonly comprising injection-site reactions, fatigue, chills, and fever (Norberg et al. 2025). Thrombotic events have also been reported. Approval was granted through priority review, with orphan drug and breakthrough therapy designations.

## Genetic disorders

Therapeutic advances in genetic disorders in 2025 span nucleoside replacement strategies, cell-based gene therapy, and viral vector–mediated gene delivery, reflecting a broadening of precision approaches for rare inherited diseases. A combination of pyrimidine nucleosides, *doxecitine and doxribtimine*, has been approved for the treatment of thymidine kinase 2 deficiency in adults and pediatric patients with symptom onset at or before 12 years of age. This marks the first approved therapy for this rare, inherited disorder, which is caused by mutations in *TK2* that impair mtDNA replication and leads to progressive neuromuscular disease (Chow et al. 2025). Treatment with pyrimidine nucleosides reduced the risk of death, stabilized or improved motor milestones, respiratory function, and feeding support and was generally well tolerated, with mostly mild adverse events reported (Domínguez-González et al. 2025). The therapy is administered orally. Elevated liver transaminase levels and gastrointestinal AE are listed as warnings and precautions. The most common AEs reported are diarrhea, vomiting, an increase in liver enzymes, and abdominal pain.

In parallel with metabolic correction strategies, cell-based gene therapies have also emerged for severe inherited skin disorders. *Prademagene zamikeracel* is the first autologous cell-based gene therapy for the treatment of recessive dystrophic epidermolysis bullosa. This is a rare connective tissue disorder caused by mutations in the *COL7A1* gene that prevent the production of functional type VII collagen, resulting in extreme skin fragility and wounds (Tang et al. 2025). Keratinocytes harvested from nonwounded patient skin are transduced ex vivo with a retroviral vector encoding full-length human COL7A1 and expanded into keratinocyte sheets. A single surgical application of *prademagene zamikeracel* sheets demonstrated improved wound healing and reduced pain compared with standard care (Tang et al. 2025). Long-term safety and efficacy were further supported by a phase 1/2a clinical trial (So et al. 2022). The therapy was generally well tolerated, with AEs including procedural pain and pruritus. Warnings include drug hypersensitivity, retroviral vector–mediated insertional oncogenesis, and transmission of infectious agents. Approval was granted with breakthrough therapy and orphan drug designations.

## Dermatology

Recent approvals in dermatology include small-molecule inhibitors designed to modulate inflammatory signaling pathways in chronic skin diseases. *Delgocitinib*, the first topical Janus kinase (JAK) inhibitor, was approved for the treatment of moderate-to-severe chronic hand eczema in adults. Concomitant use with other JAK inhibitors or systemic immunosuppressants is not recommended. Twice-daily topical application of delgocitinib demonstrated superior efficacy compared with vehicle cream over a 16-week treatment period (Bissonnette et al. 2024). Clinical studies further showed that delgocitinib was generally well tolerated and maintained efficacy for up to 52 weeks (Nakagawa et al. 2024; Gooderham et al. 2025). The most commonly reported AEs include application-site pain, paresthesia, pruritus, erythema, skin infections, leukopenia, and neutropenia. However, as a member of the JAK inhibitor class, delgocitinib carries potential class-related risks, including serious infections, nonmelanoma skin cancers, possible increased mortality in patients with cardiovascular risk factors, and lipid elevations. However, there was no clear evidence of major systemic JAK inhibitor–related risks in clinical trials. Given the class-associated safety concerns and the presence of systemic exposure following topical administration, such risks cannot be fully excluded. Therefore, approval was granted with appropriate labeling warnings.

In parallel, targeted inhibition of B cell signaling pathways has emerged as a novel approach for inflammatory skin disorders driven by mast cell activation. *Remibrutinib* is a first-in-class Bruton’s tyrosine kinase (BTK) inhibitor approved for the treatment of chronic spontaneous urticaria in adult patients who remain symptomatic despite H1 antihistamine treatment. By inhibiting BTK, remibrutinib prevents the release of histamine and other proinflammatory mediators from mast cells and basophils. Oral remibrutinib improved key symptoms, including pruritus and wheal formation, by week 12 of the treatment (Metz et al. 2025). The therapy carries a risk of bleeding, which may be increased with concomitant use of antithrombotic agents. Frequently reported AEs include nasopharyngitis, bleeding, headache, nausea, and abdominal pain. Beyond dermatology, remibrutinib is also being evaluated in clinical trials for other diseases such as Sjögren’s syndrome (Dörner et al. 2024).

## Ophthalmology

Recent approvals in ophthalmology include *acoltremon*, a first-in-class agonist of the transient receptor potential melastatin 8 (TRPM8) thermoreceptor approved for the treatment of dry eye disease. TRPM8 ion channels are expressed on cold-sensitive neurons innervating the cornea and upper eyelids and play a key role in regulating basal tear production. Ophthalmic administration of acoltremon enhances tear secretion and reduces dry eye disease symptoms (Pattar et al. 2025). The therapy was generally safe and well tolerated, with mild instillation-site pain being the most commonly reported AE (Zhou et al. 2025).

In 2025, ophthalmology has also seen progress toward disease-modifying therapies for rare retinal disorders. *Revakinagene taroretcel-lwey*, an encapsulated cell-based gene therapy, was approved to treat idiopathic macular telangiectasia (MacTel) type 2 in adults. MacTel is a rare neurodegenerative disease associated with retinal atrophy or neovascular changes that lead to progressive central vision loss (Hoy 2025b). *Revakinagene taroretcel-lwey* is administered via surgical intravitreal implantation and provides continuous retinal delivery of ciliary neurotrophic factors (CNTF), thereby slowing disease progression. Notably, the therapy has been shown to produce sustained bioactive CNTF for more than a decade, supporting its long-term therapeutic potential (Kauper et al. 2025). The therapy carries warnings for severe vision loss, infectious endophthalmitis, retinal tears or detachment, vitreous hemorrhage, implant extrusion, cataract formation, suture-related complications, and delayed dark adaptation. Common AEs include conjunctival hemorrhage, eye pain, pruritus, ocular discomfort, blurred vision, headache, dry eye, cataract progression, vitreous floaters, and iridocyclitis. *Revakinagene taroretcel* has been granted orphan drug and fast track designations.

## Other

Targeting neurokinin pathways, *tradipitant* has been approved for the prevention of motion-induced vomiting in adults. It is a substance P/neurokinin-1 receptor antagonist administered as a single oral dose on an empty stomach about 60 min prior to anticipated motion. Tradipitant effectively prevented vomiting associated with motion sickness across a range of sea conditions (Polymeropoulos et al. 2025). The therapy may impair the ability to drive or operate heavy machinery. The most common AEs are somnolence, headache, and fatigue. Beyond motion sickness, tradipitant has also been investigated in gastroparesis and atopic dermatitis (Welsh et al. 2021; Carlin et al. 2024) but not approved as of now for either indication.

## General trends and conclusions

The average number of drugs approved yearly since 2021 has been 48. Within the 45 drugs plus 4 cellular and gene therapy products approved in 2025, oncology as a field once again had the greatest share with the continuing trend for approval of ever smaller subtypes defined by specific biomarkers. The increasing biomarker-driven stratification increasingly necessitates single-arm studies, enrichment strategies, and surrogate end points, often in very small patient populations. While this enables faster access for highly selected patients, it also shifts a growing part of the evidence generation into the post-approval setting. Also continuing a trend, cardiovascular/respiratory disorders again contribute in a major way to overall approvals.

Another trend over the past 6 years has been a steady flow of first-in-indication approvals hovering around the 10% of all approvals line (Fig. 1). Not surprisingly, this largely represents approvals for orphan indications (see Table 3) as most prevalent conditions already have established medical treatment options, although not always optimal ones. Against this background, first-in-class, that is, drugs with a new mechanism of action, also remains steady with about 40% of all new approvals, whereas next-in-class remains steady at about 50%. That about half of all new approvals belong to the next-in-class groups does not mean that these are not innovative, as they may offer benefits in efficacy and/or tolerability and/or other patient-relevant parameters.

Among drug types, small molecules remain the bedrock of medical treatment, representing about 60% of all newly approved drugs (Fig. 3). Obviously, small molecules, typically suitable for oral or topical administration, have many benefits for patients (easy administration) and pharmaceutical companies (lower cost of manufacturing as compared to other drug types). Therapeutic antibodies also remain steady at about 20% of newly approved drugs. On the other hand, the last couple of years witnessed a continuous decline in newly approved peptides and proteins other than antibodies, whereas mRNA, gene, and cell-based treatments have increased, presently representing about 20% of all new approvals. This reflects that such advanced therapies often offer treatment options for targets not easily druggable by small molecules or antibodies.

The most impressive trend in recent years has been the strong performance of novel drugs for orphan indications. This obviously is a blessing for afflicted patients as many of these did not have any approved treatments. The societal downside of this trend is that based on a small expected market size for each of these drugs, the total size of eligible patients is small. As research and development costs remain unchanged, such drugs tend to be very expensive. Consequently, future discussions will need to balance rapid access to highly specialized therapies with the sustainability of healthcare systems, particularly as one-time high-cost treatments become more common. Many orphan approvals are based on platform technologies that allow repeated use of a validated modality across multiple indications. This suggests that the high proportion of orphan drugs is not only driven by unmet need but also by the strategic efficiency of such development platforms.

The lack of innovative treatments for infectious diseases has often been commented upon, mostly under the aspect of developing resistance among bacteria for existing antibiotics. Therefore, we consider it noteworthy that 2025 saw four new anti-infective treatments getting approved including two antibacterial drugs, both being first-in-class treatments. Finally, novel treatments, particularly novel oncology drugs, have traditionally come from European and US pharmaceutical companies. However, China now has a very active and innovative pharmaceutical industry. Accordingly, two of the drugs newly approved by the FDA, penpulimab and sunvozertinib, were originally developed in China and received regulatory approval there several years ago. This also reflects a changing regulatory sequencing strategy, in which first approval is increasingly sought in regional markets before subsequent FDA submission, illustrating the globalization of drug development and the growing importance of multiregional clinical programs. We expect this trend to become even more important in coming years.

## Data Availability

Not applicable.

## Abbreviations

ADC: Antibody drug conjugate
AE: Adverse event
BTK: Bruton’s tyrosine kinase
CSF1: Colony-stimulating factor 1
EGFR: Epidermal growth factor receptor
FDA: US Food and Drug Administration
HER2: Human epidermal growth factor receptor 2
JAK: Janus kinase
MEK: Mitogen-activated protein kinase kinase
NSCLC: Non–small cell lung cancer
PD-1: Programmed death receptor-1
siRNA: Small interfering RNA
RSV: Respiratory syncytial virus
